# Prognostic value of the geriatric nutritional index in colorectal cancer patients undergoing surgical intervention: A systematic review and meta-analysis

**DOI:** 10.3389/fonc.2022.1066417

**Published:** 2022-11-23

**Authors:** Yiqing Mao, Jiarong Lan

**Affiliations:** ^1^ Department of Gastrointestinal Surgery, Huzhou Central Hospital, Affiliated Central Hospital Huzhou University, Huzhou, China; ^2^ School of Basic Medical Sciences, Zhejiang Chinese Medical University, Hangzhou, China; ^3^ Department of Medicine, Huzhou Traditional Chinese Medicine Hospital Affiliated to Zhejiang Chinese Medical University, Huzhou, China

**Keywords:** nutrition, prognosis, survival, complications, cancer

## Abstract

**Background:**

We reviewed the literature to assess the prognostic ability of the geriatric nutritional risk index (GNRI) for patients with colorectal cancer (CRC) undergoing curative surgery.

**Methods:**

The online databases of PubMed, CENTRAL, ScienceDirect, Embase, and Google Scholar were searched for articles reporting the relationship between GNRI and outcomes in CRC patients. English language studies were searched up to 28^th^ April 2022.

**Results:**

Ten studies with 3802 patients were included. Meta-analysis indicated that patients with low GNRI had significantly poor overall survival (HR: 2.41 95% CI: 1.72, 3.41 I^2 =^ 68%) and disease-free survival (HR: 1.92 95% CI: 1.47, 2.49 I^2 =^ 49%) as compared to those with high GNRI. The meta-analysis also indicated a significantly higher risk of complications with low GNRI as compared to high GNRI (HR: 1.98 95% CI: 1.40, 2.82 I^2 =^ 0%). The results did not change on subgroup analysis based on study location, age group, GNRI cut-off, and sample size.

**Conclusion:**

Current evidence indicates that GNRI can be a valuable prognostic indicator for CRC patients undergoing surgical intervention. Patients with low GNRI have poor overall and disease-free survival and a higher incidence of complications. Clinicians could use this simple indicator to stratify patients and formulate personalized treatment plans.

**Systematic Review Registration:**

https://www.crd.york.ac.uk/prospero/, identifier (CRD42022328374).

## Introduction

Cancer has become the most common cause of mortality worldwide. Amongst the numerous subtypes, colorectal cancer(CRC) ranks the 2^nd^ most prevalent cancer in females and 3^rd^ most common malignancy amongst men around the globe (1). The prevalence has been high in Asian populations but a large number of patients are also being detected in Western regions and developing nations (2). CRC is known to have a predilection for the older age group as the median age of diagnosis is reported to be 67 years (3). Western data indicate that of the approximately 140,000 confirmed cases of CRC detected in 2018, around 60% were elderly with an age of >65 years. Furthermore, older adults accounted for almost 70% of mortality cases in the same period (4). Identification of modifiable risk factors for poor prognosis can aid in appropriate treatment planning and improve the long-term overall survival (OS) and disease-free survival (DFS) of CRC patients. Of the numerous risk factors identified, malnutrition has been one of the most prominent and well-defined factors associated with poor survival after CRC (5). However, there has been no consensus in the literature on how to measure malnutrition to best assess the prognosis of such patients (6). Several measurement indices like the body mass index, bodyweight loss, serum albumin levels, psoas muscle area, mini-nutritional assessment, prognostic nutritional index, and controlling nutritional status score have been used to quantify malnutrition in a cancer patient (7, 8). Since >50% of patients with gastrointestinal (GI) cancer suffer from malnutrition, there is a need for an easy to calculate and robust malnutrition indicator which has a good prognostic ability (7). The Geriatric Nutritional Risk Index (GNRI) is a simple malnutrition screening tool estimated from serum albumin levels and ideal body weight (9). It has been used in literature to assess the prognosis of patients undergoing percutaneous coronary interventions, and hemodialysis as well as for those with heart and respiratory diseases (10–13). The tool has also received attention in the field of oncology with several studies reporting its use for different cancers (14–16). Recently, Xie et al (17) have reviewed the ability of GNRI to predict the prognosis of patients with GI malignancies. In a pooled analysis of nine studies, the authors reported that patients with low GNRI had a significantly higher risk of complications and poor long-term survival as compared to those with high GNRI. An important limitation of their review was patients with different GI cancer were pooled in a single meta-analysis. Over the past few years, several authors (18–20) have reported their experience with the use of GNRI for CRC patients but there has been no consolidated review to examine the available evidence. Given this deficiency in literature, we present the results of the first systematic review and meta-analysis examining the prognostic ability of GNRI for CRC patients undergoing curative surgical resection.

## Material and methods

### Search and eligibility

The review was pre-registered on PROSPERO (No CRD42022328374). The PRISMA recommendations were used during the reporting of the review (21). A detailed search on the online databases of PubMed, CENTRAL, ScienceDirect, Embase, and Google Scholar was conducted for articles reporting the prognostic ability of GNRI for CRC patients. The search was last done on 28^th^ April 2022. Two reviewers were independently involved in the search which was restricted to English-language publications only. The search terms were; “colorectal cancer”, “rectal cancer”, “geriatric nutritional risk index”, “prognosis”, “nutrition”, and “survival”. The search was conducted by combining the search terms with Boolean operators “OR” and “AND”. Details can be found in **Supplementary Table 1**. The search results combined for initial titles and abstract screening. Only studies relevant to the review were extracted and matched against the eligibility criteria. The entire procedure involved two reviewers working independently.

The eligibility criteria were all types of studies reporting the relationship between GNRI and outcomes of CRC patients undergoing curative resections. The outcomes were OS, DFS, and/or complications. We excluded studies 1) not reporting data for CRC patients separately 2) not on patients undergoing surgical intervention 3) not reporting any of the relevant outcomes 4) studies with duplicate data. If there were two studies from the same center conducted during the same period the article with the largest sample was to be included.

In the final stage, the full-text articles were screened based on the eligibility criteria, and those fulfilling the same were included. Any differences in study selection were resolved by discussion. Lastly, we also hand-searched the reference list of included studies and previous reviews to look for any missed articles.

### Data management

Using an Excel spreadsheet the following data were extracted from the included studies: Details of study authors, publication year, study location, study type, inclusion criteria, the cut-off for GNRI, sample size, age, gender, carcinoembryonic antigen (CEA) levels, location of cancer (colon or rectal), tumor invasion, presence of lymph node metastasis, use of adjuvant therapy, follow-up and outcomes. The outcomes assessed in the review were OS, DFS, and complications. We assessed the risk of bias using the Newcastle-Ottawa scale (NOS) (22).

### Statistical analysis

The prognostic ability of GNRI was reported as multivariable-adjusted hazard ratios (HR) by most studies. These were extracted and combined in a random-effects model to calculate the total effect size as HR with 95% confidence intervals (CI). We assessed inter-study heterogeneity using the I^2^ statistic. Publication bias was examined by visual inspection of funnel plots and a sensitivity analysis was also performed. Sub-group analysis was carried out based on study location (Japanese vs non-Japanese), age group included (≥65 years, ≥75 years, and others), GNRI cut-off (98 and others), and sample size (>300 and <300). Results were reported in tabular format. Funnel plots, sensitivity analysis, and subgroup analysis was not conducted for complication rates due to limited data. For studies not reporting outcomes as adjusted ratios, a descriptive analysis was undertaken. The data analysis was conducted using “Review Manager” (RevMan, version 5.3; Nordic Cochrane Centre [Cochrane Collaboration], Copenhagen, Denmark; 2014).

## Results

**Figure 1:**
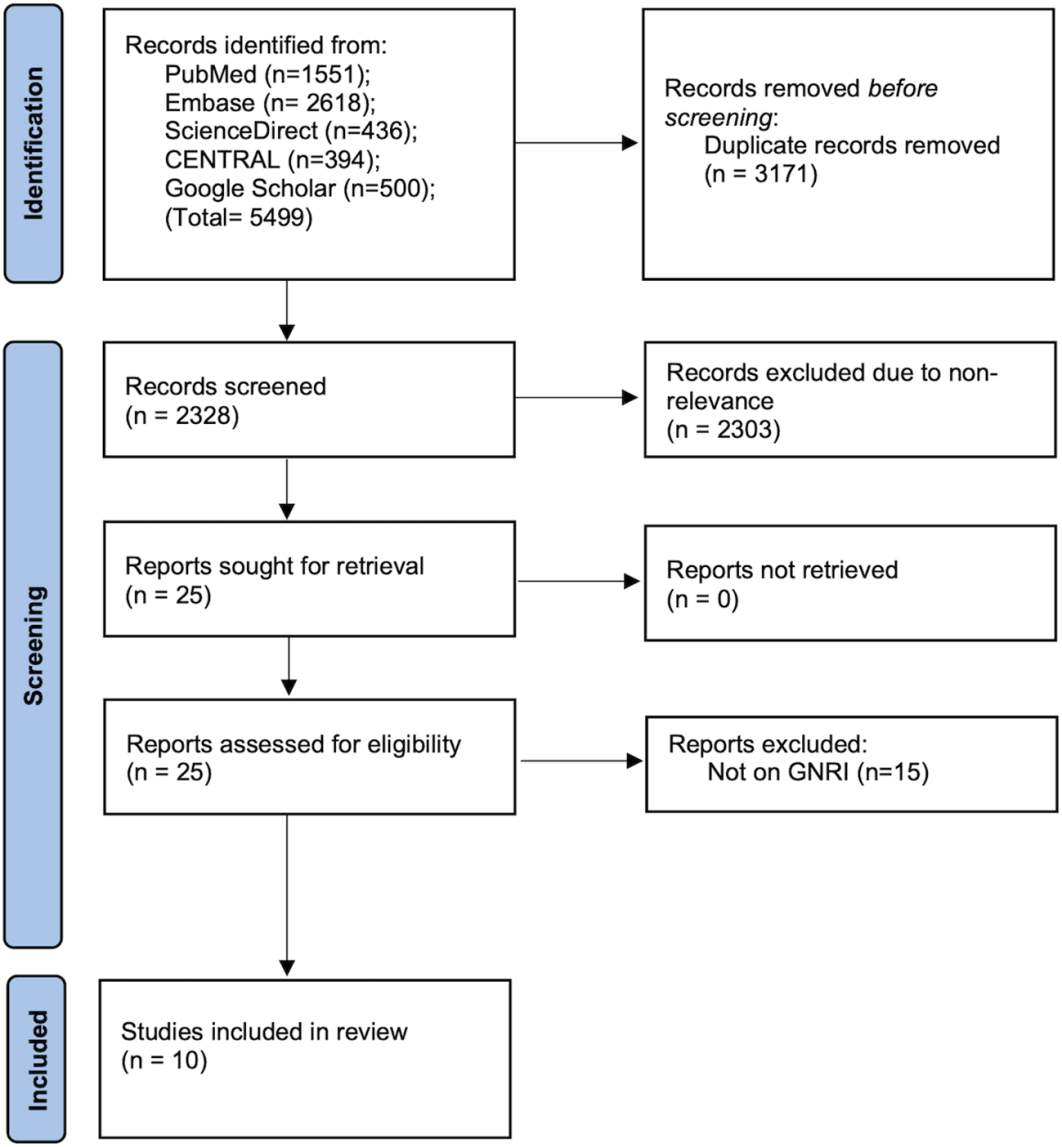
Details of literature search in the PRISMA flow-chart.

The initial search resulted in 5499 articles (**Figure 1**). After deduplication, 2328 articles were screened by the reviewers. 25 of these were selected for further analysis. Finally, ten studies were deemed eligible for inclusion in the review (18–20, 23–29).

All included studies were retrospective observational studies conducted in Asian countries (**Table 1**). Most of them were carried out in the Japanese population. One was from Taiwan and another study was from China. The number of participants in the studies ranged from 80 to 1206. The total pooled sample size was 3802 patients. Most studies included all elderly patients with CRC undergoing curative resection. However, there were some exceptions. One study included patients only with locally advanced rectal cancer, while another included individuals only with stage Tis/T1 CRC undergoing endoscopic submucosal dissection, and one study included those with CRC liver metastasis. The percentage of male patients ranged from 44 to 79.6% in the included studies while the proportion of patients with high CEA ranged from 28.9 to 48.8%. The distribution of colon and rectal cancer varied across included studies. Details on tumor invasion, lymph node metastasis, and the use of adjuvant therapy were not reported by all studies. All studies had a follow-up of more than 1 year. The NOS score ranged from 7 to 8.

**Table 1:** Details of included studies.

Study	Study Location	Patients	Cut-off of GNRI	Groups	Sample size	Mean/Median age (years)	Male gender (%)	CEA, ≥5 (%)	Location	T3-T4 stage (%)	Lymph node metastasis (%)	Adjuvant therapy (%)	Follow-up	NOS
Yagyu 2022 (29)	Japan	Elderly patients (≥75 years) with stage II CRC	93.465	High Low	147 201	81 83	46.9 45.8	NR	C 78.9%, R 21.1% C 79.6%, R 20.4%	9.5* 24.4	NR	12.9 10.9	Up to 5 years	8
Hayama 2022 (28)	Japan	Elderly patients (≥65 years) with stage I-III CRC	101.1	High Low	207 51	NR	43.4 44	34.5% 36%	C 19.7%, R 80.3% C 9.8%, R 90.2%	35.4 4	32.4 38	NR	Median 1214 days	8
Doi 2022 (27)	Japan	Patients with stage I-III CRC	98	High Low	190 139	71.4 76.9	44.7 45.3	32.8% 44.9%	NR	55.8 77.7	32.1 31.7	NR	Median 32.1 months	8
Liao 2021 (26)	Taiwan	Elderly patients (≥75 years) with stage I-III CRC	98	High Low	662 544	79.5 81.6	57.7 53.5	30.1% 40%	C 62.2%, R 37.8% C 73.7%, R 26.3%	75.4 86.4	39.9 42	NR	Median 60.7 months	8
Kato 2021 (24)	Japan	Elderly patients (≥75 years) with Tis/T1 CRC undergoing ESD	96.3	NR	691	NR	NR	NR	NR	NR	NR	NR	Median 41-46 months	7
Kataoka 2021 (25)	Japan	Elderly patients (≥65 years) with CRC	98	High Low	127 127	75.3 74.9	51.2 52.8	48.8% 48%	C 65.4%, R 34.6% C 66.1%, R 33.9%	NR	NR	NR	Up to 5 years	8
Ide 2021 (20)	Japan	Patients with locally advanced rectal cancer undergoing CRT	104.25	High Low	55 38	NR	72.7 73.7	47.3% 60.5%	R 100% R 100%	NR	NR	42 23	Median 60.03 months	8
Tang 2020 (23)	China	Elderly patients (≥65 years) with CRC	98	High Low	117 113	NR	54.7 79.6	37.6% 35.4%	C 45.3%, R 54.7% C 54.9%, R 45.1%	68.4 69	NR	NR	Median 61 months	8
Sasaki 2020 (19)	Japan	Elderly patients (≥65 years) with CRC	98	High Low	176 137	NR	70.5 56.2	28.9% 35.4%	C 77.8%, R 33.2% C 74.5%, R 35.5%	49.4 48.2	26.7 29.9	NR	Median 60.5 months	8
Iguchi 2020 (18)	Japan	Patients with stage CRC and synchronous liver metastasis	98	High Low	50 30	62.4 65.5	52 60	NR	NR	88 90	NR	75.5 69	Mean 1545 days	8

*only T4 stage.

CRC, colorectal cancer; CEA, Carcinoembryonic antigen; GNRI, Geriatric Nutritional Risk Index; C, colon; R, rectal; NR, not reported; NOS, Newcastle Ottawa scale.

**Figure 2:**
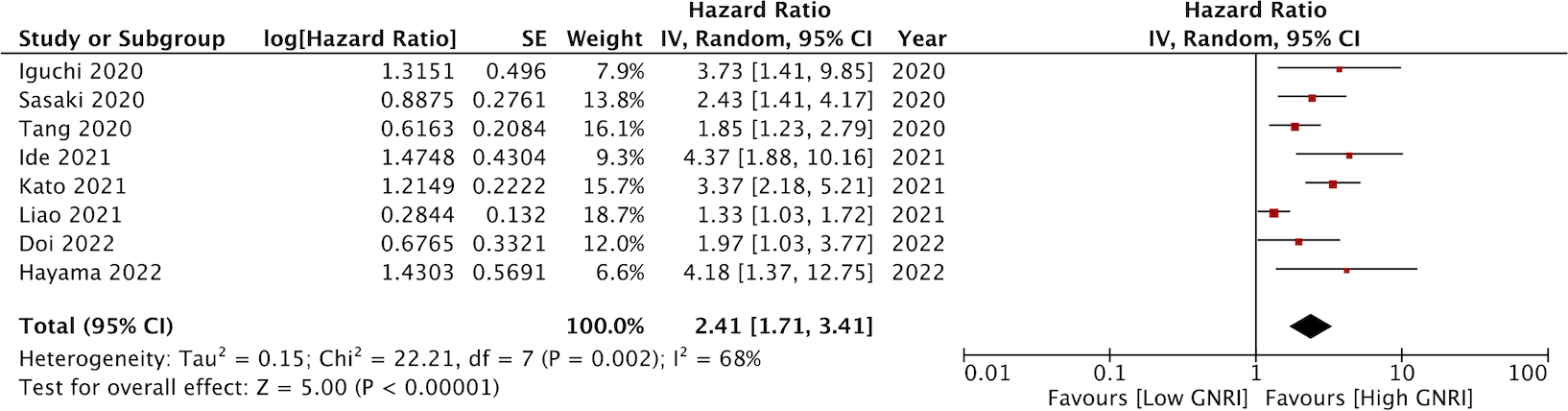
Forest plot of the prognostic ability of GNRI for OS in CRC patients.

**Figure 3:**
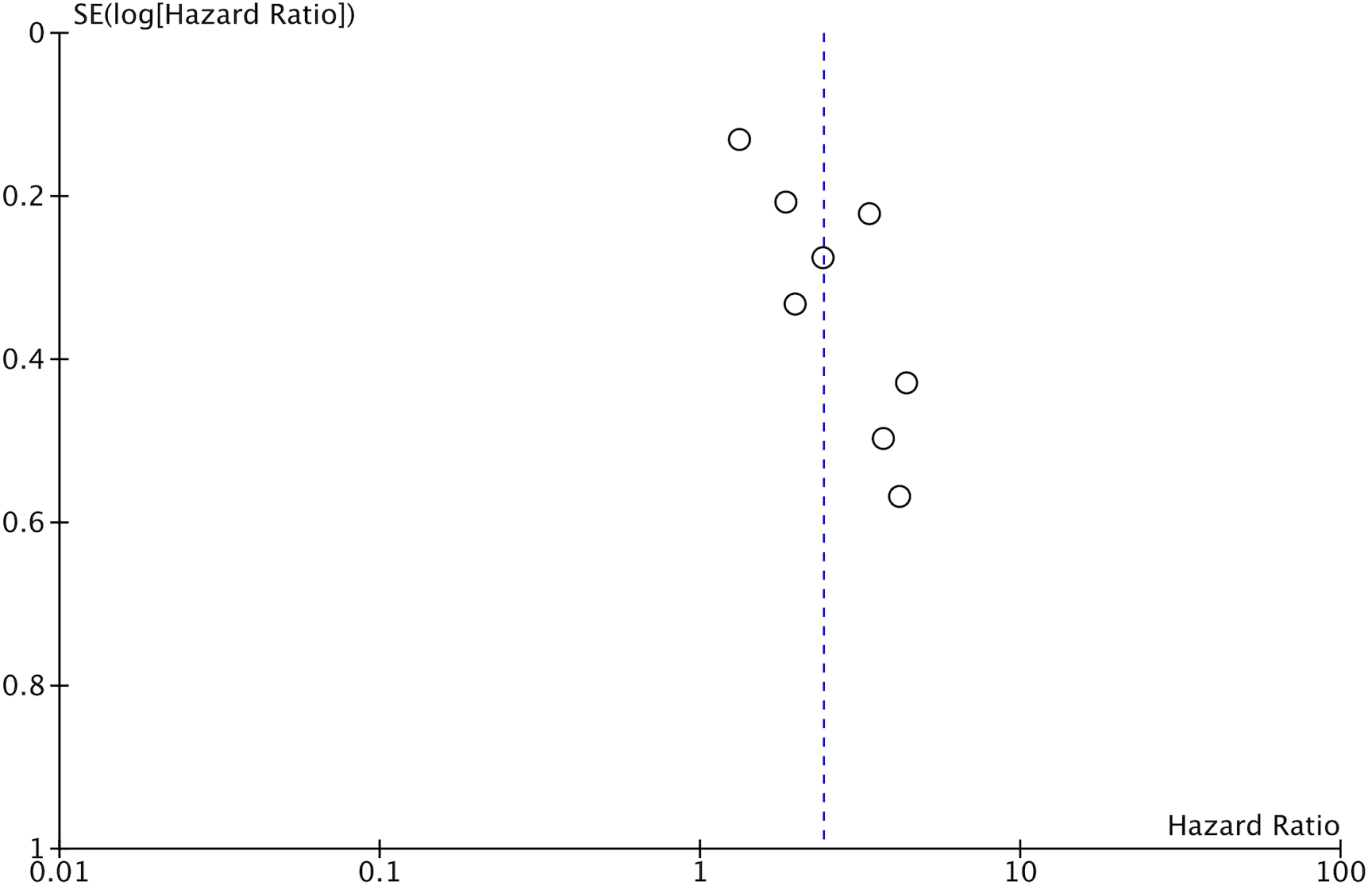
Funnel plot for the meta-analysis on the prognostic ability of GNRI for OS in CRC patients.

Eight studies reported data on OS. Meta-analysis indicated that patients with low GNRI had significantly poor OS as compared to those with high GNRI (HR: 2.41 95% CI: 1.72, 3.41 I^2 =^ 68%) (**Figure 2**). The results remained the same on the sequential exclusion of studies during the sensitivity analysis. We did not note any publication bias on the visual inspection of the funnel plot (**Figure 3**).

**Figure 4:**
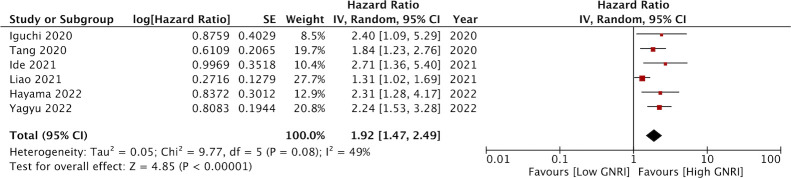
Forest plot of the prognostic ability of GNRI for DFS in CRC patients.

**Figure 5:**
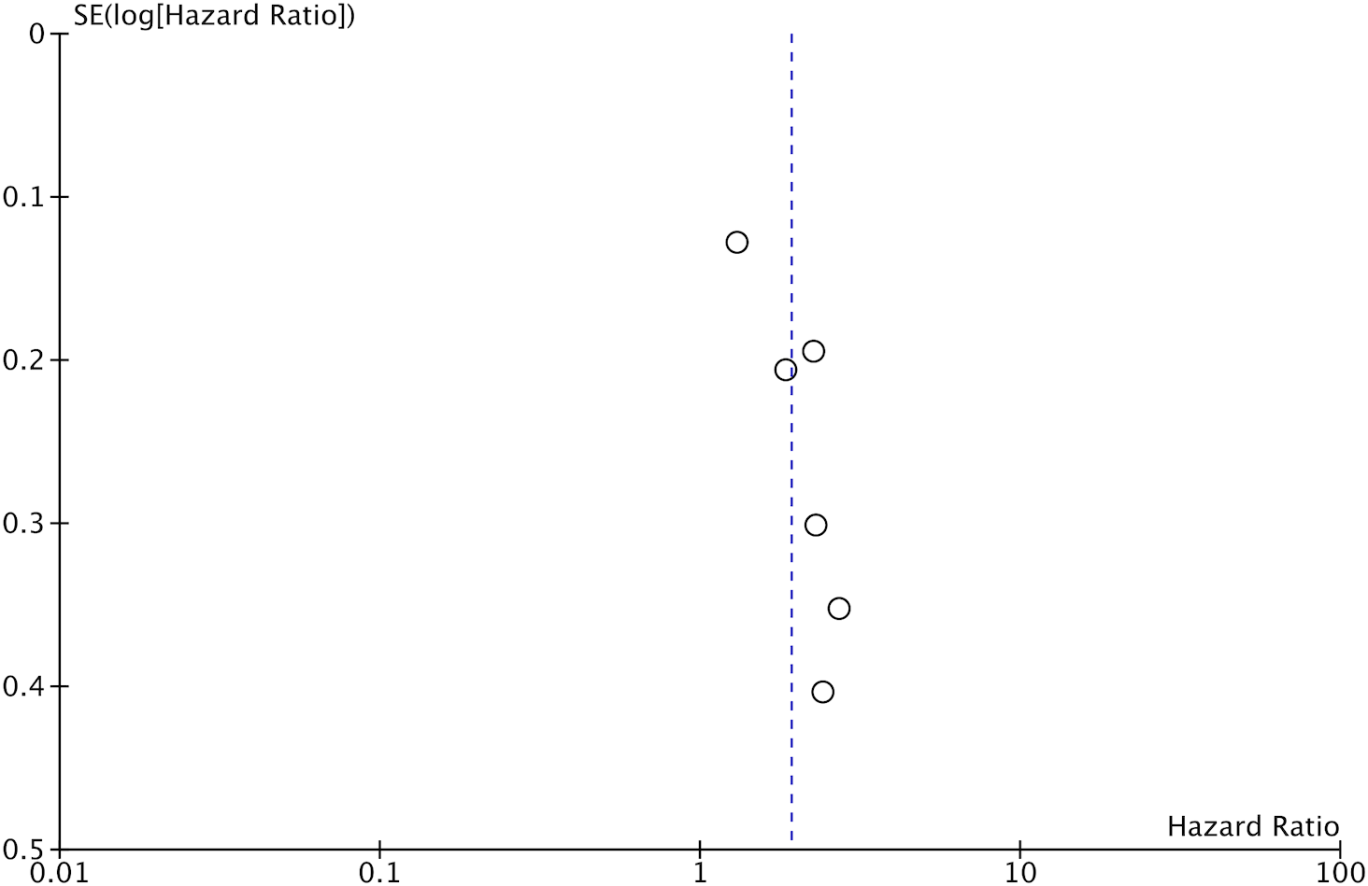
Funnel plot for the meta-analysis on the prognostic ability of GNRI for DFS in CRC patients.

Data on DFS was available only from six studies. On pooled analysis, we noted that low GNRI was a significant predictor of poor DFS in CRC patients (HR: 1.92 95% CI: 1.47, 2.49 I^2 =^ 49%) (**Figure 4**). There was no change in the significance of the results on sensitivity analysis. There was no evidence of publication bias noted on the funnel plot (**Figure 5**).

**Figure 6:**
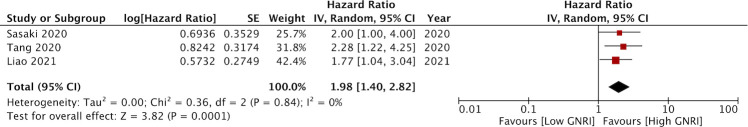
Forest plot of the prognostic ability of GNRI for complications in CRC patients.

Only three studies assessed the prognostic ability of GNRI for predicting complications. Meta-analysis indicated a significantly higher risk of complications with low GNRI as compared to high GNRI (HR: 1.98 95% CI: 1.40, 2.82 I^2 =^ 0%) (**Figure 6**).

The results of subgroup analysis for OS and DFS are reported in **Table 2**. We noted that subgroup analysis for the outcome OS based on study location, GNRI cut-off, and sample size did not change the significance of the results. However, GNRI was not predictive of OS on a pooled analysis of two studies including only those with ≥75 years of age. The results of the outcome DFS did not change on subgroup analysis based on study location, age group, GNRI cut-off, and sample size.

**Table 2:** Subgroup analysis.

Variable	Groups	Number of studies	Hazard ratio
OS			
Study location	Japanese Non-Japanese	6 2	2.98 (95%CI: 2.29, 3.89 I^2 =^ 0%) 1.51 (95%CI: 1.10, 2.07 I^2 =^ 45%)
Age group included	≥65 years only ≥75 years only Others	3 2 3	2.17 (95%CI: 1.57, 3.00 I^2 =^ 3%) 2.08 (95%CI: 0.84, 5.17 I^2 =^ 92%) 2.93 (95%CI: 1.74, 4.93 I^2 =^ 21%)
GNRI cut-off	98 Others	5 3	1.86 (95%CI: 1.37, 2.54 I^2 =^ 50%) 3.62 (95%CI: 2.51, 5.22 I^2 =^ 0%)
Sample size	>300 <300	4 4	2.11 (95%CI: 1.29, 3.45 I^2 =^ 79%) 2.91 (95%CI: 1.75, 4.86 I^2 =^ 42%)
DFS			
Study location	Japanese Non-Japanese	4 2	2.34 (95%CI: 1.79, 3.08 I^2 =^ 0%) 1.50 (95%CI: 1.08, 2.07 I^2 =^ 49%)
Age group included	≥65 years only ≥75 years only Others	2 2 2	1.98 (95%CI: 1.42, 2.77 I^2 =^ 0%) 1.68 (95%CI: 1.00, 2.84 I^2 =^ 81%) 2.57 (95%CI: 1.53, 4.32 I^2 =^ 0%)
GNRI cut-off	98 Others	3 3	1.60 (95%CI: 1.16, 2.19 I^2 =^ 42%) 2.34 (95%CI: 1.75, 3.12 I^2 =^ 0%)
Sample size	>300 <300	2 4	1.68 (95%CI: 1.00, 2.84 I^2 =^ 81%) 2.14 (95%CI: 1.61, 2.83 I^2 =^ 0%)

OS, overall survival; DFS, disease free survival; GNRI, Geriatric Nutritional Risk Index; CI, confidence intervals.

Only one study by Kataoka et al (25) did not report outcomes as adjusted ratios and hence could not be included in the meta-analysis. In their study, the authors used propensity score matching to compare data of patients with low and high GNRI (cut-off 98). Patients with low GNRI had significantly poor OS (p=0.002), DFS (p=0.006), and a higher rate of complications (p=0.001) as compared to those with high GNRI.

## Discussion

Cancer patients have a high prevalence of malnutrition and muscle wasting which is known to negatively affect survival and increase the length of hospital stays. Indeed, the catabolic and physiological impact of cancer cachexia escalates the nutritional and energy requirement of the individual but it is seldom met due to inadequate dietary intake and reduced physical activity (5). Malnutrition is further exacerbated in GI malignancies due to additional factors like malabsorption, obstructive syndrome, and diarrhea (7). Research has shown that malnutrition is unexpectedly high in patients with GI malignancies with clinicians recognizing only 1 out of 4 patients with malnutrition leading to inadequate pretreatment nutritional support and poor outcomes. This illustrates the fact that nutritional screening is of utmost importance even when the patient shows no overt signs of malnutrition (30). One of the limitations of various nutritional screening tools is their varying sensitivity and specificities. Ideally, the screening tool should be simple, brief, inexpensive, with high sensitivity and good specificity (31).

In this context, the GNRI was developed by Bouillanne et al. in 2005 as a simple tool to predict outcomes in elderly patients using albumin and body weight data (9). Since then the tool has been used to predict outcomes in a variety of patients (10–13). Several meta-analysis studies have reported the predictability of GNRI for various cancer subtypes. Wang et al (32) in a meta-analysis of eight studies have shown that low GNRI is associated with poor OS (HR: 1.99, 95% CI: 1.68-2.35) and DFS (HR = 2.34, 95% CI: 1.11-4.95) in patients with non-small cell lung cancer. In another recent meta-analysis, Yu et al (33) compiled data from 14 studies and noted that low pretreatment GNRI predicted poor OS (HR = 1.47, 95% CI: 1.33-1.63) and DFS (HR = 1.69, 95% CI: 1.24-2.31) in patients with esophageal cancer. Individual studies have shown that GNRI could predict outcomes in patients with head and neck cancer, renal cancer, pancreatic cancer, and gastric cancer (14, 16, 34, 35). However, since each cancer subtype is different, it is important that the predictability of GNRI is established for CRC as well.

We conducted a detailed literature search to recognize ten studies with a total of 3802 cancer patients undergoing curative surgical resection for CRC. This provided a more homogenous group of patients undergoing the same primary treatment unlike the previous meta-analysis wherein patients with different GI malignancies undergoing different treatments were included (17). On pooled analysis, it was seen that patients with low GNRI had a 2.4 times increased risk of the poor OS as compared to those with high GNRI. Secondly, patients with low GNRI had 1.9 times increased risk of recurrence as compared to those with high GNRI. We also noted that low GNRI was significantly associated with higher rates of complications, albeit with only three studies in the meta-analysis. On examination of all three forest plots of our meta-analysis, it can be seen that the direction of the result was consistent across all studies only with varying 95% CI. None of the studies noted a non-significant association between low GNRI and outcomes in CRC patients. There was little evidence of publication bias and the survival results maintained their significance on sensitivity analysis. The results were robust and thereby increase the validity of our conclusions.

An important limitation of the meta-analysis was the moderate heterogeneity in the meta-analysis of OS and DFS. This could be due to several known and unknown variables like sample size, study location, patient demographics, baseline stage of CRC, treatment protocols, and cut-off used for GNRI. Based on the availability of data we divided the studies into separate groups based on sample size, study location, age group included, and the cut-off for GNRI only to note no change in the significance of the results. The exception was the outcome of OS in the subgroup of studies including only patients aged ≥75years. The overall effect size was 2.08 with a 95% CI of 0.84, 5.17. The non-significant results could be due to the small number of studies in the analysis as the 95% CI was wide with the lower end very close to 1 and the upper end indicating a 5 times increased risk of poor OS.

If the GNRI has to be incorporated into clinical practice, a well-established cut-off is needed to segregate patients into low and high GNRI groups. Most of the studies in our review as well as in literature (15, 33) have used the cut-off of 98 for classifying patients into those with low and high GNRI. Nevertheless, there has been no consensus and other cut-offs have been used by studies based on receiver operating curve analysis of population-specific data. In our subgroup analysis, we noted that the results were the same for studies using a cut-off of 98 or any other for assessing the prognosis of CRC patients. Future studies should focus on GNRI cut-off in different populations to arrive at a common figure for clinical practice.

The good prognostic ability of GNRI could be due to its combined use of two important markers of malnutrition: albumin and body weight (9). Low serum albumin levels have been congruous with malnutrition and hypoalbuminemia has been associated with poor wound healing, infections, and reduced survival in cancer patients. Serum albumin has an immunomodulatory role with low levels leading to reduced cell-mediated immunity against cancer cells (5). Hypoalbuminemia causes reduced macrophage activation and granuloma formation which may promote surgical site infections and other complications in CRC patients (6). Hu et al (5) in a study on 30676 CRC patients have found a statistically significant association between low albumin levels and postoperative complications like venous thromboembolism, surgical site infections, pneumonia, septic shock, prolonged ventilator use, blood transfusion, return to the operating room, stroke, and re-intubation in CRC patients. Secondly, the GNRI uses a ratio of current body weight to ideal body weight as a marker of the body mass index (BMI) of the patients. Cancer patients with low BMI are at an increased risk of poor survival (36). Thus, it can be postulated that the combination of albumin and body weight increases the ability of the GNRI to predict prognosis in cancer patients.

There are several strengths to our review. It is the first meta-analysis to aggregate evidence on the role of GNRI in predicting outcomes in CRC patients. We attempted to include a homogenous population of patients undergoing surgical intervention. The validity of the results was tested by sensitivity and different subgroup analyses.

Nevertheless, there are some limitations as well. Not all studies provided data for all three outcomes. Hence, the number of studies in the meta-analysis was less than 10. Secondly, not all studies were of large sample size and this may have reduced the power of our analysis. Thirdly, there was some heterogeneity in the study population included in the studies. Some included only patients with T1 stage while another included patients with liver metastasis. The effect of such variation could be assessed only by sensitivity analysis and not by subgroup analysis. Fourthly, all studies were on Asian populations with most studies from Japan. Thus the results cannot be generalized to other populations.

## Conclusions

Current evidence indicates that GNRI can be a valuable prognostic indicator for CRC patients undergoing surgical intervention. Patients with low GNRI have poor OS, DFS, and a higher incidence of complications. Clinicians could use this simple indicator to stratify patients and formulate personalized treatment plans. Further studies with a larger sample size are required to validate the results in non-Asian populations and obtain the most optimal cut-off to predict outcomes.

## Data availability statement

Publicly available datasets were analyzed in this study. The original contributions presented in the study are included in the article/**Supplementary Material**. Further inquiries can be directed to the corresponding author.

## Author contributions

YM and JL conceived and designed the study. YM and JL were involved in literature search and data collection. YM analyzed the data. JL wrote the paper. and JL reviewed and edited the manuscript. All authors contributed to the article and approved the submitted version.

## Conflict of interest

The authors declare that the research was conducted in the absence of any commercial or financial relationships that could be construed as a potential conflict of interest.

## Publisher’s note

All claims expressed in this article are solely those of the authors and do not necessarily represent those of their affiliated organizations, or those of the publisher, the editors and the reviewers. Any product that may be evaluated in this article, or claim that may be made by its manufacturer, is not guaranteed or endorsed by the publisher.
