# Supplementary material for: Prognostic value of the geriatric nutritional index in colorectal cancer patients undergoing surgical intervention: A systematic review and meta-analysis
Source: Front Oncol. 2022 Nov 23;12:1066417. doi: 10.3389/fonc.2022.1066417 (PMC9743169; doi:10.3389/fonc.2022.1066417)
Supplement: Supplementary file 1 [file DataSheet_1.pdf]

Supplementary Table 1: Search strategy

Query	Search Details
(((colorectal cancer[Title/Abstract]) OR (rectal cancer[Title/Abstract])) AND (survival[Title/Abstract])) AND (nutrition)	("colorectal cancer"[Title/Abstract] OR "rectal cancer"[Title/Abstract]) AND "survival"[Title/Abstract] AND ("nutrition s"[All Fields] OR "nutritional status"[MeSH Terms] OR ("nutritional"[All Fields] AND "status"[All Fields]) OR "nutritional status"[All Fields] OR "nutrition"[All Fields] OR "nutritional sciences"[MeSH Terms] OR ("nutritional"[All Fields] AND "sciences"[All Fields]) OR "nutritional sciences"[All Fields] OR "nutritional"[All Fields] OR "nutritionals"[All Fields] OR "nutritions"[All Fields] OR "nutritive"[All Fields])
(((colorectal cancer[Title/Abstract]) OR (rectal cancer[Title/Abstract])) AND (prognosis)) AND (nutrition)	("colorectal cancer"[Title/Abstract] OR "rectal cancer"[Title/Abstract]) AND ("prognosis"[MeSH Terms] OR "prognosis"[All Fields] OR "prognoses"[All Fields]) AND ("nutrition s"[All Fields] OR "nutritional status"[MeSH Terms] OR ("nutritional"[All Fields] AND "status"[All Fields]) OR "nutritional status"[All Fields] OR "nutrition"[All Fields] OR "nutritional sciences"[MeSH Terms] OR ("nutritional"[All Fields] AND "sciences"[All Fields]) OR "nutritional sciences"[All Fields] OR "nutritional"[All Fields] OR "nutritionals"[All Fields] OR "nutritions"[All Fields] OR "nutritive"[All Fields])
((colorectal cancer) OR (rectal cancer)) AND (geriatric nutritional risk index)	("colorectal neoplasms"[MeSH Terms] OR ("colorectal"[All Fields] AND "neoplasms"[All Fields]) OR "colorectal neoplasms"[All Fields] OR ("colorectal"[All Fields] AND "cancer"[All Fields]) OR "colorectal cancer"[All Fields] OR ("rectal neoplasms"[MeSH Terms] OR ("rectal"[All Fields] AND "neoplasms"[All Fields]) OR "rectal neoplasms"[All Fields] OR ("rectal"[All Fields] AND "cancer"[All Fields]) OR "rectal cancer"[All Fields])) AND (("geriatric"[All Fields] OR "geriatrics"[MeSH Terms] OR "geriatrics"[All Fields]) AND ("nutrition s"[All Fields] OR "nutritional status"[MeSH Terms] OR ("nutritional"[All Fields] AND "status"[All Fields]) OR "nutritional status"[All Fields] OR "nutrition"[All Fields] OR "nutritional sciences"[MeSH Terms] OR ("nutritional"[All Fields] AND "sciences"[All Fields]) OR

	"nutritional sciences"[All Fields] OR "nutritional"[All Fields] OR "nutritionals"[All Fields] OR "nutritions"[All Fields] OR "nutritive"[All Fields]) AND ("risk"[MeSH Terms] OR "risk"[All Fields]) AND ("abstracting and indexing"[MeSH Terms] OR ("abstracting"[All Fields] AND "indexing"[All Fields]) OR "abstracting and indexing"[All Fields] OR "index"[All Fields] OR "indexed"[All Fields] OR "indexes"[All Fields] OR "indexing"[All Fields] OR "indexation"[All Fields] OR "indexations"[All Fields] OR "indexe"[All Fields] OR "indexer"[All Fields] OR "indexers"[All Fields] OR "indexs"[All Fields]))
